# Developmental defects of enamel and dental anomalies in children with skin diseases

**DOI:** 10.1007/s00784-025-06326-0

**Published:** 2025-04-14

**Authors:** Alev Eda Okutan, Ayşe Deniz Yücelten, Ali Menteş

**Affiliations:** 1 Private Practice, Istanbul, Turkey; 2 Department of Dermatology, Faculty of Medicine, Marmara University, Istanbul, Turkey; 3 Department of Pediatric Dentistry, Faculty of Dentistry, Marmara University, Istanbul, Turkey

**Keywords:** MIH, Developmental defects of enamel, Hypodontia, Taurodontism, Skin diseases, Genodermatoses

## Abstract

**Objectives:**

The ectodermal origin of enamel and skin suggests a potential link between dermatological conditions and dental anomalies. This study aimed to evaluate the prevalence of developmental defects of enamel (DDE) and other dental anomalies in pediatric patients with skin diseases.

**Materials and methods:**

This cross-sectional study included 71 pediatric patients (aged 4–14) diagnosed with chronic skin diseases or genodermatoses, along with 41 age- and sex-matched controls. Enamel defects were assessed using both the Modified DDE Index and the Molar-Incisor Hypomineralization (MIH) Index, while panoramic radiographs were analyzed for hypodontia, taurodontism, short root anomaly, and primary failure of eruption.

**Results:**

DDE-not-related MIH was significantly higher in the study group (*p* = 0.01). Additionally, other dental anomalies were significantly more prevalent in the study group (*p* = 0.03), particularly among patients with genodermatoses (*p* < 0.001). Ectodermal dysplasia and epidermolysis bullosa exhibited the highest prevalence of dental anomalies.

**Conclusion:**

Our findings suggest a possible association between skin diseases, particularly genodermatoses, and an increased prevalence of dental anomalies. These results highlight the importance of early dental screening and multidisciplinary care for children with dermatological conditions.

**Clinical relevance:**

Understanding the dental implications of skin diseases can aid clinicians in early diagnosis and preventive strategies, improving oral health outcomes for affected children.

## Introduction

Amelogenesis is a complex process that begins with the secretion of enamel matrix proteins, followed by mineralization, and finally, the maturation of the entire structure [1]. Factors affecting amelogenesis and causing developmental defects of enamel (DDE) are traditionally classified as either hereditary or acquired [2]. Causes of DDE include environmental intoxications such as fluoride and dioxins, trauma, localized infections, irradiation, chemotherapy, infectious diseases, perinatal and postnatal problems, and malnutrition [3]. Demarcated opacities in the first permanent molars, a type of DDE, have been well-known and regarded as a separate entity by pediatric dentists since they were first defined by Weerheijm in 2001 as molar incisor hypomineralization (MIH) [4]. In recent years, epidemiological studies on enamel defects have focused on MIH. Despite numerous investigations into potential factors, the etiology of MIH remains unclear. It is likely that MIH is caused not by a single factor but by a combination of multiple factors [2].

Developmental alterations of teeth affect not only the enamel but also the structure, number, size, and shape of teeth [5]. From initiation to the final basic features such as shape, size, and structure, teeth are typical examples of organs in which genes predominantly determine the progress of development, while environmental factors play a minor role [6]. Every disease reflects the interactions between genes and the environment [7]. In monogenic disorders, a single mutation can lead to a specific phenotype [7]. However, the spectrum and degree of manifestations depend on numerous environmental factors [7]. The common embryologic neural origin of the ectoderm includes the epidermal layer of the skin and the amelodentinal (the enamel and dentine) components of the teeth, resulting in various conditions affecting both the skin and dentition to different degrees [8].

Dermatitis, also known as eczema, is a chronic relapsing inflammatory skin condition that causes dry skin, redness, and itchiness. Its etiology is not clear, but recent research indicates that its pathogenesis is complex, resulting from a combination of genetic and environmental factors that induce skin barrier dysfunction, cutaneous and systemic immune dysregulation, skin microbiota dysbiosis, and a strong genetic influence. Specific types of dermatitis are more common in certain age groups. For example, atopic dermatitis (AD) is much more common in children than in adults [9].

Psoriasis is one of the most frequent chronic and inflammatory skin diseases in childhood. Polygenic susceptibility and environmental triggers are believed to be involved in the pathogenesis of psoriasis. Like AD, psoriasis presents with scaly and dry skin [9].

Nevus is a nonspecific medical term for a visible, circumscribed, chronic lesion of the skin or mucosa. The term nevus is applied to a number of conditions caused by neoplasias and hyperplasias of melanocytes, as well as a number of pigmentation disorders, both hypermelanotic (containing increased melanin, the pigment responsible for skin color) and hypomelanotic (containing decreased melanin) [9].

Mastocytosis encompasses a group of rare clinical entities that are characterized by abnormal growth and, usually, a low accumulation of clonal and morphologically abnormal mast cells within one or more organs. Childhood-onset mastocytosis is generally detectable during the first year of life. This type of mastocytosis is most often restricted to the skin [9].

Morphea, or localized scleroderma, is an inflammatory disease that primarily affects the skin and underlying tissues, leading to sclerosis of the affected tissues. Besides genetic predisposition, there are environmental triggers such as traumatic factors, radiation, medications, and infections [9].

The skin is the organ of the human body that is most commonly involved in monogenic diseases. More than one-third of all genetic diseases affect the integument. Currently, more than 350 genodermatoses have been identified with functional insights [9].

Genetically inherited skin diseases affect not only the skin but also the oral tissues. For example, in ectodermal dysplasia (ED), dental anomalies like hypodontia are a core clinical feature [9].

The ichthyoses are a large, heterogeneous group of skin cornification disorders. They can be inherited or acquired and result in defective keratinocyte differentiation and abnormal epidermal barrier formation. The resultant skin barrier dysfunction leads to increased transepidermal water loss and inflammation. Disordered cornification is clinically characterized by skin scaling with various degrees of thickening, desquamation (peeling), and erythema (redness) [9].

Xeroderma pigmentosum is one of the rare genetically inherited skin diseases that causes sensitivity to ultraviolet radiation from sunlight, resulting in pigmentation changes in the skin, and can lead to skin cancers [9]. Additionally, epidermolysis bullosa (EB) is a very rare disease caused by mutations of proteins in the dermo-epidermal junction. The disease has four phenotypic subgroups: Epidermolysis Bullosa Simplex (EBS), Junctional Epidermolysis Bullosa (JEB), Dystrophic Epidermolysis Bullosa (DEB), and Kindler Syndrome (KEB). Junctional EB is notable for its effects on teeth, known as amelogenesis imperfecta (AI). AI may be a phenotypic enamel manifestation in syndromes like JEB. All the genes related to JEB are involved in ameloblast cell adhesion, which is critical for normal enamel mineralization [9].

Albinism includes a group of genetically inherited autosomal recessive conditions typically characterized by a congenital reduction or absence of melanin pigment biosynthesis, which gives the natural color to the skin, iris, and hair. Traditionally, albinism has been classified according to clinical phenotype, with the two main categories being oculocutaneous albinism (OCA) types 1, 2, and 3, and ocular albinism [9].

Our literature search showed that the overall relationship between dermatological conditions and the developmental anomalies of teeth is not fully explored. Considering that the skin shares a common embryological background with enamel, the factors that cause any skin disorder may also cause developmental anomalies in the teeth [10]. Hence, the aim of the study was to evaluate the occurrence of dental anomalies (DDE, MIH, hypodontia, hyperdontia, taurodontism, root malformations, short root anomaly, and primary failure of eruption) among pediatric patients with skin diseases.

## Materials and methods

### Sample size calculation

GPower statistical software (v.3.1.9.6., Universitaet Kiel, Kiel, Germany) was used to calculate the sample size. The calculation was based on the findings of the study by Hernandez et al., which showed a statistically significant relationship between AD and MIH [22]. The researchers investigated the relationship between different diseases and MIH and reported that MIH was observed significantly more in the population with AD (OR: 90.9; CI: 33.4–247.1; *p* < 0.001). Accordingly, for the two-tailed sample size calculation of the present study, the significance level alpha was set at 0.05, and the critical z level was determined to be 1.95 at a power level of 98%. In line with these parameters, it was concluded that the sample size should be at least 97, with at least 45% in the study group. Considering a 10% dropout risk, the minimum sample size was determined to be 107.

### Study design

We conducted the present comparative cross-sectional observational study during September 2017–May 2018. A total of 254 pediatric patients were examined by a pediatric dermatologist (ADY) and a pediatric dentist (AEO) at the relevant institution within the specified time period. However, 120 cases (47.2%) were excluded due to repeated follow-up visits with the same patients, leaving a total of 134 unique patients. Additionally, 51 patients (20.1%) or their parents declined participation or were unwilling to visit the dental clinic for further evaluation, reducing the number to 83 patients. Furthermore, 12 patients (4.7%) were uncooperative during the intraoral examination, making it impossible to obtain reliable data. As a result, a final total of 71 patients (28.0%) met the inclusion criteria and were included in the study group.

### Inclusion criteria

- Pediatric patients aged 4–14 who applied to the dermatology clinic where chronic and genetic skin diseases are followed up. Only patients with an official diagnosis confirmed by a dermatology specialist (ADY) and followed up in the dermatology clinic were included.
- Children or their parents who accepted to visit pediatric dentistry clinic.
- Children who were cooperative for clinical examination and radiographic imaging.
- Children whose legal guardians provided informed consent.

### Exclusion criteria

- Patients with acute dermatological conditions.
- Patients not between the ages of 4 and 14.
- Children who did not comply with the implementation of the procedures required for the investigation.

Forty-one healthy children who applied to Marmara University Department of Pediatric Dentistry for the first time, matched by age and gender with the study group, were designated as the control group. The health status of the patients, indicating the absence of skin diseases, was confirmed based on their medical records and parents’ self-proclamation.

### Clinical examination

Patients eligible for inclusion in this study were identified during the clinical examinations at the Marmara University Department of Dermatology Pediatric Follow-up Clinic while they were being examined for their dermatological condition. The patients’ diagnoses and medications were verified and confirmed by a dermatology specialist (ADY), and the archive records of Marmara University Dermatology Department were scanned for the patients’ past history.

Although the first intraoral examinations were performed at the dermatological outpatient clinic, the second intraoral examinations were conducted in the dental settings of the pediatric dentistry clinic. These examinations were performed by a single trained examiner (AEO) under the same dental unit and reflector, with a smooth-surfaced standard mouth mirror (#5) and tactile inspection with a standard dental probe (PCP11). We evaluated enamel defects with the Modified Developmental Defects of Enamel Index (mDDE Index), which is the preferred index in many epidemiological studies [11]. This index includes normal, demarcated opacities (white/cream or yellow/brown), diffuse opacities (diffuse lines, diffuse patchy, diffuse confluent, confluent/patchy, + and loss of enamel), and hypoplasia (pits, missing enamel, or any other defects). Elfrink et al. stated that intraoral dental photographs can be used to evaluate DDE [12]. Thus, to facilitate repeated evaluations, dental photographs were taken. An intraoral digital camera (Canon EOS 750D) and macro lens (Canon EF 100 mm f/2.8 L IS USM, Tokyo, Japan) fixed on the camera with an owl bracket were used to take intraoral photographs. The camera was used in “Auto” mode for each shot.

Because of the deficiencies of the mDDE Index in recording atypical restorations or caries, post-eruptive enamel breakdown, MIH-related tooth loss, and amelogenesis imperfecta, we used the MIH Index for the repeated examinations [13]. Teeth affected by MIH and DDE-related MIH were evaluated using the criteria approved by the European Academy of Paediatric Dentistry (EAPD) in 2015 [14]. These criteria include no visible enamel defects, enamel defects that are not related to MIH (diffuse opacities, hypoplasia, amelogenesis imperfecta, hypomineralization defect), white/creamy and yellow/brown demarcated opacities, post-eruptive enamel breakdown, atypical MIH-specific restorations and caries, tooth loss due to MIH, and unscored conditions [14]. The intra-examiner Kappa values of the examiner who diagnosed MIH and DDE-not-related MIH ranged from 0.83 to 0.89. The high degree of agreement was retained during re-examination periods.

We had some limitations for taking photos of all patients. For ichthyosis patients, we couldn’t use a dental retractor because of their angular cheilitis. Additionally, we couldn’t take photos of a four-year-old child because of her dental anxiety. Due to these limitations, we used the initial evaluation’s mDDE Index scores and renamed them to the MIH Index.

Panoramic radiographs were taken using a panoramic X-ray machine (Planmeca Promax, Planmeca, Finland), and dental anomalies observed in the radiographs (hypodontia, hyperdontia, taurodontism, short root anomaly) were recorded.

The diseases of the children in the study group were grouped as genodermatoses (Ectodermal Dysplasia, Epidermolysis Bullosa, Xeroderma Pigmentosum, Ichthyosis, Albinism) and chronic skin diseases (Dermatitis, Psoriasis, Morphea, Mastocytosis, Nevus) under the guidance of the dermatology specialist (ADY). Then, statistical analyses were performed to compare the obtained data between the control group and the study group and its subgroups.

### Statistical analyses

SPSS version 21.0 software (IBM, Chicago, Illinois, USA) was used for statistical analysis. Descriptive analyses of numerical variables were calculated as the mean and standard deviation. The chi-square and Fisher’s exact tests were used for the analysis of categorical variables. The Shapiro-Wilk test was used for normality analysis. We used the Student’s t-test or Mann-Whitney U test to compare the numerical data of two groups based on the normality analysis. Values equal to or less than 0.05 (*p* ≤ 0.05) were interpreted as statistically significant.

## Results

The study group consisted of 71 pediatric patients diagnosed with various dermatological conditions. The distribution of diagnoses was as follows: dermatitis (*n* = 34), nevus (*n* = 9), psoriasis (*n* = 8), epidermolysis bullosa (*n* = 7), ichthyosis group of disorders (*n* = 5), ectodermal dysplasia (*n* = 2), xeroderma pigmentosum (*n* = 2), morphea (*n* = 2), mastocytosis (*n* = 1), and albinism (*n* = 1).

The mean age of the study group and the control group was 9.05 ± 2.47 and 8.51 ± 2.66, respectively (*p* = 0.19). There were 40 (56%) girls and 31 (44%) boys in the study group, and 21 (51%) girls and 20 (49%) boys in the control group (*p* = 0.6) Table 1.

**Table 1:** Demographic data of the study and control groups

		Study group (*n* = 71)	Control group (*n* = 41)	*p*
Age		9.05 ± 2.47	8.51 ± 2.66	0.19^‡^
Sex	Girl	40 (56%)	21 (51%)	0.6*
	Boy	31 (44%)	20 (49%)	

^‡^: Mann Whitney U Test, *: Student T Test

Table 2 presents the distribution of dental anomalies among patients with skin diseases. Dermatitis was the most frequently observed condition (47.9%), with MIH (29.5%) and DDE-not-related MIH (11.8%) being the most common enamel defects. Other dental anomalies were observed in 17.5% of dermatitis patients, including hypodontia (8.8%) and primary failure of eruption (2.9%) Table 2.

**Table 2:** Skin diseases in the study group and the types and percentages of anomalies seen in these diseases

Skin Diseases Group	Diseases	MIH	DDE not related MIH	Other anomalies				
				Total	Hypodontia	Taurodontism	Short Root Anomaly	Primary Failure of Eruption
Chronic skin diseases	Dermatitis (*n* = 34)	10 (29.5%)	4 (11.8%)	3 (17.5%)	3 (8.8%)			1 (2.9%)
	Psoriasis (*n* = 8)	2 (25%)	1 (12.5%)	1 (12.5%)	1 (12.5%)			
	Morphea (*n* = 2)	1 (50%)						
	Mastocytosis (*n* = 1)		1 (100%)					
	Nevus (*n* = 9)	2 (22.2%)	3 (33%)					
Genodermatoses	Ectodermal Dysplasia (*n* = 2)			2 (100%)	2 (100%)			
	Epidermolysis Bullosa (*n* = 7)		7 (100%)	3 (43%)		1 (14.3%)		3 (42.9%)
	Xeroderma Pigmentosum (*n* = 2)		1 (50%)					
	Ichthyosis (*n* = 5)		1 (20%)	2 (40%)			1 (20%)	1 (20%)
	Albinism (*n* = 1)	1 (100%)						

Among genodermatoses, ectodermal dysplasia and epidermolysis bullosa showed the highest prevalence of dental anomalies. All patients with ectodermal dysplasia (100%) presented with hypodontia, while taurodontism (14.3%) and primary failure of eruption (42.9%) were prominent in epidermolysis bullosa cases. Ichthyosis was associated with short root anomaly (20%) and primary failure of eruption (20%) Table 2.

MIH was detected in 23% (*n* = 16) of the study group. Although this rate was approximately twice the 12% MIH rate in the control group (*n* = 5), there was no statistically significant difference between the groups (*p* = 0.17) Table 3. DDE-not-related MIH was found to be statistically significantly higher in the study group than in the control group (*p* = 0.01). Additionally, other dental anomalies (hypodontia, taurodontism, short root anomaly, and primary failure of eruption) were found to be statistically significantly higher in the study group than in the control group (*p* = 0.03).

**Table 3:** Evaluation of anomaly types percentages in study and control groups

Defect Types	Study group (*n* = 71)		Control group (*n* = 41)		*p* ^+^
	+	-	+	-	
MIH	16 (23%)	55 (77%)	5 (12%)	36 (88%)	0.17
DDE not related MIH	18 (25%)	53 (75%)	3 (7%)	38 (93%)	**0.01**
Other Anomalies	11 (15%)	60 (85%)	1 (2%)	40 (98%)	**0.03**

^+^: Ki Square Test

We evaluated the study group in two subgroups: chronic skin diseases (*n* = 54) and genodermatoses (*n* = 17) Table 4. Although there was no statistically significant difference between subgroups in terms of MIH, a statistically significant difference was found in the genodermatoses group for DDE-not-related MIH (*p* = 0.002) and other dental anomalies (*p* < 0.001). Table 5 shows the comparison of the genodermatoses group with the control group. In this table, DDE-not-related MIH and other dental anomalies show high statistical significance in favor of the genodermatoses group Table 5.

**Table 4:** Evaluation of anomaly types percentages in study groups subgroups

Defect types	Genodermatosis (*n* = 17)		Chronic Skin diseases (*n* = 54)		*p* ^+^
	+	-	+	-	
MIH	1 (6%)	16 (94%)	15 (28%)	39 (72%)	0.059
DDE not related MIH	9 (53%)	8 (47%)	9 (17%)	45 (83%)	**0.002**
Other Anomalies	7 (41%)	10 (59%)	4 (7%)	50 (93%)	**< 0.001**

^+^: Ki Square Test

**Table 5:** Evaluation of anomaly types in genodermotosis group and control group

Defect types	Genodermatosis (*n* = 17)		Control group (*n* = 41)		*p* ^+^
	+	-	+	-	
MIH	1 (6%)	16 (94%)	5 (12%)	36 (88%)	0.47
DDE not related MIH	9 (53%)	8 (47%)	3 (7%)	38 (93%)	**< 0.001**
Other Anomalies	7 (41%)	10 (59%)	1 (2%)	40 (98%)	**< 0.001**

^+^: Ki Square Test

Table 6 shows the evaluation of dental anomalies in the chronic skin disease group and the control group. In this table, MIH and DDE-not-related MIH percentages are statistically significantly higher in the chronic skin disease group than in the control group (*p* = 0.004, *p* = 0.2, respectively) Table 6.

**Table 6:** Evaluation of anomaly types in chronic skin diseases group and control group

Defect types	Chronic Skin diseases (*n* = 54)		Control group (*n* = 41)		*p* ^+^
	+	-	+	-	
MIH	15 (28%)	39 (72%)	5 (12%)	36 (88%)	**0.004**
DDE not related MIH	9 (17%)	45 (83%)	3 (7%)	38 (93%)	**0.02**
Other Anomalies	4 (7%)	50 (93%)	1 (2%)	40 (98%)	0.08

^+^: Ki Square Test

## Discussion

This study encompasses the dental findings of pediatric patients visiting dermatology clinics. We believe it is clinically significant as it collectively evaluates the oral and dental health of children with dermatological problems. Interactions of multiple genes, epigenetic factors, and environmental influences not only affect the development of the skin but also the shape of individual teeth, groups of teeth, and the dentition as a whole [15]. To date, more than 200 genes involved in embryonic development, morphogenesis, and differentiation of tooth processes have been identified [16].

There are several dermatologic disorders that have a genetic etiology or a genetic predisposition, called “genodermatoses” [17]. Most of the genodermatoses cause anomalies that affect the size, number, shape, or structure of the tooth [8].

One of the genodermatoses is ectodermal dysplasia (ED), and in many EDs, dental defects such as hypodontia and oligodontia of the primary and permanent teeth represent a core clinical feature [18]. The oral findings of the ED patients in the current study are consistent with the literature.

Current information regarding dental anomalies in epidermolysis bullosa (EB) is based on case reports, most of which describe patients with generalized enamel hypoplasia [19–21], one with failure of tooth eruption [19], one with a localized enamel defect [22], and one with amelogenesis imperfecta [23]. In the current study, in addition to generalized DDE, amelogenesis imperfecta, and failure of tooth eruption, hypodontia and taurodontism were also detected.

The oral manifestations of xeroderma pigmentosum (XP) are mainly related to the occurrence of malignant tumors in the lips, tongue, and buccal mucosa [9]. In the current study, DDE in XP was revealed for the first time, but there was no finding of oral malignancy in our patients as in the literature.

Dental anomalies in ichthyosis patients are based on case reports. There is limited knowledge about the oral manifestations of ichthyosis. In some studies, the presence of supernumerary teeth [24], hypodontia [25], and DDE [26] were reported. Basel-Vanagaite et al. described conical (deciduous) teeth or notched and pitted permanent teeth in three individuals with ichthyosis and hypotrichosis [27]. In the current study, in addition to hypodontia and DDE, short root anomaly was revealed for the first time.

Varied dental features have been reported in albinism patients. In a case of oculocutaneous albinism (OCA), the patient had an upper maxillary lateral incisor showing features of both dens invaginatus (dens in dente) and dens evaginatus [28]. In another case, enamel hypoplasia has been reported in brothers with OCA in both primary and permanent dentition [9]. In the current study, MIH was found in an albinism patient; the oral findings of the albinism patient did not match the literature.

In the current study, dental anomalies in the genodermatoses group were compared with both the chronic skin disease group and the healthy control group. DDE-not-related MIH, such as amelogenesis imperfecta, and other dental anomalies, such as hypodontia, were found to be significantly higher in the genodermatoses group, in line with the literature. This confirms that such anomalies are frequently seen in genetically-based diseases.

The concept of multifactorial etiology is more prominent in chronic skin diseases. In the current study, dermatitis constituted 63% of the chronic skin disease group. There are studies in the literature investigating the relationship between atopic dermatitis and dental defects. In the study on the effect of atopic dermatitis (AD) on dentition published by Tan et al. (2022), enamel hypoplasia (33%) and hypodontia (13%) were reported [29]. Sodré in 2020 found 17.4% of the demarcated opacities in AD patients [30]. However, both of them were cross-sectional studies in which they did not have a control group to compare their results with a healthy population. In the study by Periqua about dental manifestations of AD in 2017, it was shown that anatomical dental abnormalities, including agenesia, hypoplasia, black-stain, tooth fusion, dental ankylosis, and odontogenic tumors, were seen in 13/90 (14.4%) of AD patients [31]. Hernandez revealed, for the first time, a statistically significant relationship between AD and the presence of MIH. In Hernandez’s study, it was found that 45% of children with MIH had AD [12]. The results of the dermatitis patients in the current study also support the literature.

Although there are well-documented reports of psoriatic lesions appearing on oral soft tissues (lips, tongue, palate, buccal mucosa, and gingiva), there is only one case report focused on oral hard tissues, describing a patient with psoriasis who has oligodontia and DDE [32]. Similar to the results of this case report, half of the psoriasis patients in the current study had dental defects (25% MIH, 12.5% DDE-not-related MIH, and 12.5% hypodontia).

Oral mucosa and dental involvement have only been rarely described in morphea [33]. In a survey of the reported 25 cases of morphea associated with oral and dental manifestations, besides the typical cutaneous features, the patients presented unerupted teeth, root development defects, root atrophy, and short roots [33]. In the current study, there was no involvement of morphea lesions in the mouth in either of our morphea patients, and, contrary to the literature, there was no root problem. However, the current study revealed MIH in a morphea patient for the first time.

It has been reported that more than 50% of patients with mastocytosis have bone pathology. Apart from a case report documenting mast cell infiltration in the jawbone [34], no other reports regarding the oral findings of this disease havebeen found in the literature.

of this disease have been found in the literature. Due to the lack of sufficient data in the literature and the small number of mastocytosis patients in the current study, it is not possible to discuss the results.

Reports describing intraoral anomalies associated with nevi are few. In a review, dental anomalies observed in 13 patients with oral linear epidermal nevus were reported as odontoma in one, and hypoplasia and hypodontia in the other [35]. In both cases, linear lesions were intraorally placed. Many inherited and acquired diseases of the skin or mucosa manifest themselves in a linear configuration. The embryological basis of the distribution pattern of these lines remains, so far, an enigma, although it has been reported that they may represent visible embryonic migration paths of clones of ectodermal cells that carry genetic disorders [36]. In some of our patients, the lesions were in a linear configuration, but in none of our patients were the lesions adjacent to oral tissues. In the current study, 55% of patients with nevi had different types of enamel defects, but none of them had any dental anomalies like hypodontia, as reported in the literature.

According to the current study, while DDE-not-related MIH and dental anomalies predominated in the genodermatoses group, the opposite was the case in chronic skin diseases, where MIH was found to be significantly higher.

The progression of dermatitis group diseases in the world and the perspective of researchers on this disease are not much different from the perspective of pedodontists on MIH. In prevalence studies, results differ for both diseases in different parts of the world [37, 38]. Interestingly, both diseases are not easily diagnosed [39, 40]. It is thought that environmental and genetic effects play a role in the etiology of both diseases [2, 9]. In recent years, these diseases have attracted more attention from researchers, and null mutations in the filaggrin (FLG) gene have been established as a strong genetic risk factor for AD [41]. Therefore, future studies with larger sample sizes of patients with dermatologic diseases are necessary to confirm and expand upon our findings, providing a better understanding of the dental characteristics associated with these disorders and to understand what affects our teeth and skin separately or together.

### Limitations and strength of this study

This study has several limitations that should be acknowledged:

The sample size for some specific conditions, such as albinism and xeroderma pigmentosum, was limited, restricting the ability to draw definitive conclusions about these individual diseases. However, despite these small numbers, the study found statistically significant differences in the prevalence of DDE-not-related MIH and other dental anomalies between genodermatoses and chronic skin diseases (*p* = 0.002 and *p* < 0.001, respectively) Table 4. While this suggests a potential association, further studies with larger cohorts are required to confirm these findings and establish causality.

The grouping of skin diseases into genodermatoses and chronic skin diseases was based on their etiology and clinical characteristics, as recommended by dermatology specialists (ADY). Genodermatoses are genetically inherited disorders affecting the skin and ectodermal derivatives [42], while chronic skin diseases are persistent inflammatory conditions with unclear etiology and immune dysregulation [43]. The study did not exclude diseases based on low patient numbers to maintain methodological balance, as similar numbers existed in both categories. The primary objective was to evaluate overall trends in dental anomalies within these two broader disease groups, rather than drawing conclusions from individual rare conditions.

The etiology of DDE is multifactorial, involving both genetic and environmental influences, as highlighted in the EAPD 2021 guidelines [44]. As a cross-sectional study, it was inherently impossible to account for all potential etiological factors due to the retrospective nature of data collection. The study’s primary focus was on the impact of dermatological diseases on dental anomalies; therefore, other potential etiological factors (e.g., early childhood illnesses, allergies, systemic medication use) were excluded to maintain a focused research question. Certain medications, such as antibiotics, corticosteroids, and chemotherapeutic agents, may contribute to enamel defects. However, since the study focused on dermatological conditions rather than systemic medication exposure, a detailed medication history was not included in the analysis. Additionally, as the study population consisted entirely of children with dermatological diseases, many had a history of early childhood illnesses or medication use, making it difficult to isolate medication effects separately from their primary diagnosis.

Despite these limitations, this study provides valuable insights into the relationship between skin diseases and dental anomalies, emphasizing the importance of oral health monitoring in pediatric dermatology patients. Unlike previous studies, this research examined children who were referred to the dermatology clinic rather than dental clinics, meaning that they were not selected based on pre-existing oral complaints. This reduces selection bias and allows for a more unbiased assessment of dental anomalies in children with skin diseases. Therefore, the study provides a unique perspective on the oral health of pediatric dermatology patients who might otherwise not seek dental care.

Future studies with larger, longitudinal datasets could further explore these associations and clarify the underlying mechanisms.

## Conclusions

The current study confirmed a connection between MIH and chronic skin diseases and suggests a possible association between genodermatoses, DDE-not-related MIH, and other dental anomalies. However, due to the limited number of cases for certain conditions, further large-scale studies are needed to validate these findings.

Children with skin diseases may be at higher risk for poor oral health and esthetic concerns. To better understand the underlying mechanisms and develop effective management strategies for dental anomalies in this population, further research is essential.

Dental screening and management should be integrated into the overall health care plan of patients with skin diseases, and clinicians should be aware of the potential oral health implications associated with these conditions.

## Data Availability

The datasets generated during and/or analyzed during the current study are available from the corresponding author on reasonable request.
